# Chemical Contamination Pathways and the Food Safety Implications along the Various Stages of Food Production: A Review

**DOI:** 10.3390/ijerph18115795

**Published:** 2021-05-28

**Authors:** Kgomotso Lebelo, Ntsoaki Malebo, Mokgaotsa Jonas Mochane, Muthoni Masinde

**Affiliations:** 1 Department of Life Sciences, Central University of Technology, Private Bag X20539, Bloemfontein 9301, South Africa; nmalebo@cut.ac.za (N.M.); mmochane@cut.ac.za (M.J.M.); 2 Centre for Sustainable SMART Cities, Central University of Technology, Private Bag X20539, Bloemfontein 9301, South Africa; emasinde@cut.ac.za

**Keywords:** food safety, heavy metals, persistent organic pollutants, regulatory strategies

## Abstract

Historically, chemicals exceeding maximum allowable exposure levels have been disastrous to underdeveloped countries. The global food industry is primarily affected by toxic chemical substances because of natural and anthropogenic factors. Food safety is therefore threatened due to contamination by chemicals throughout the various stages of food production. Persistent Organic Pollutants (POPs) in the form of pesticides and other chemical substances such as Polychlorinated Biphenyls (PCBs) have a widely documented negative impact due to their long-lasting effect on the environment. This present review focuses on the chemical contamination pathways along the various stages of food production until the food reaches the consumer. The contamination of food can stem from various sources such as the agricultural sector and pollution from industrialized regions through the air, water, and soil. Therefore, it is imperative to control the application of chemicals during food packaging, the application of pesticides, and antibiotics in the food industry to prevent undesired residues on foodstuffs. Ultimately, the protection of consumers from food-related chemical toxicity depends on stringent efforts from regulatory authorities both in developed and underdeveloped nations.

## 1. Introduction

Food control is a function carried out globally due to its public health importance. However, efforts in the enforcement and implementation of legislation regarding international codes and standards remain a challenge [1,2,3]. This challenge is further exacerbated by the ever-rising human population which is estimated to reach nine billion by the year 2050, thus creating a demand for increased food production [4]. The mass production of food and high demand contributes significantly to non-conformance to best practices and legal requirements. As a result, food control is prominent and various strategies are devised to alleviate the impact of non-compliance. Analytical tools for food control have been developed over the years; however, research suggests that none of these tools provide an exclusive and unique solution in food safety [1,5]. However, it must be noted that some organizations have established early warning analytical systems to detect food safety risks on time and to improve the efficacy of surveillance in the food industry [6]. The beginning of the food production chain provides various food safety challenges. In food processing, chemicals may already be present in raw food. This happens due to advances in food science and the continued use of agrochemicals. The use of pesticides and fertilizers increases the risk of food contamination significantly [7], and this risk is observed predominantly in food industries. Agricultural land situated in the vicinity of heavy industries can introduce contamination through the water, soil, and air. This can ultimately cause a double burden of contamination due to the cumulative effect of agrochemicals and industrial pollutants [7,8]. Possible contamination from toxic natural and industrial pollutants can be tested using a variety of techniques in the food industry. The major techniques with multi-element capability in the determination of contaminants are: inductively coupled plasma atomic emission spectrometry (ICP: OES) and graphite furnace atomic absorption spectrometry [8,9,10], inductively coupled plasma mass spectrometry, flame atomic absorption spectrometry, and cold vapor atomic absorption spectrometry [5].

In a quest to promote the safety of food globally, regulatory bodies have advocated for the declaration of food ingredients and contents through labelling and responsible marketing. This is in line with global trade markets and transparency [11]. A challenge of note is that national regulations and guidelines do not always make provisions for all chemical contaminant thresholds. This is because other chemical substances are legal in one country but prohibited in other parts of the world. According to Ahmad and co-workers, it is advisable to create a global agro-business chemical control program to ensure regulatory compliance [1]. This will ensure that participating nations understand the chemical safety standards expected in international food trade. Moreover, this comes with benefits such as consumer confidence, as stipulated in the Codex Alimentarius. This review paper provides an international view on current trends on food contaminants and how they impact the environment and human health. It further focuses on the chemical contamination pathways along the various stages of food production until the food reaches the consumer.

## 2. Food Contamination along the Food Production Chain

**Figure 1:**
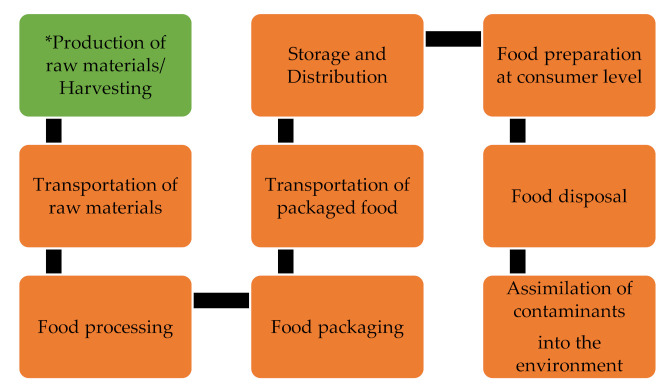
Key steps in food production, processing (farm-to-fork) till disposal. The figure is produced by authors.

Food contaminants of chemical nature can be typically classified into four categories, namely; natural toxins, environmental contaminants, agrochemical residues, and food process toxicants together with intentionally added chemicals [1,12,13,14]. Therefore, the food production chain poses an intrinsic and extrinsic risk of contamination [15,16]. As shown in Figure 1, there are various levels of food production and each stage has points where contamination can be introduced. The classifications of food contamination points are summarized below:

### 2.1. Transportation

Food can be contaminated during transportation as a result of both diesel and petrol engine vehicles through exhaust systems that emit excessive carbon monoxide. In developing countries, transportation systems and logistics management systems are not as efficient regarding the shortening of distances when transporting food [17]. This increases the likelihood of unwanted substances settling on the foodstuffs. The contaminants can settle on the packaging material or directly on the food. The most commonly checked gases for permeation on packaging material are oxygen, carbon dioxide, and water vapor. Therefore, other undetected compounds may infiltrate the barriers in the packaging [5]. Moreover, not all barriers applied to foodstuffs are effective against organic compounds. Increased efforts are encouraged during the transportation process to limit food contamination exposure levels.

### 2.2. Cleaning Agents

Cleaning agents in the food industry play a pivotal role in food safety. Amongst chemical sanitisers, there are compounds such as peracetic acid, hydrogen peroxide, and sodium hypochlorite which are favored in deep cleaning in the food industry [18,19]. Disinfectants and most cleaning agents contain harmful compounds that have a pungent smell and corrosive properties. Notwithstanding their role in the deep cleaning of surfaces and the environment at large, they can easily be introduced into food through mishandling and unsafe practices, leading to residual toxicity [19,20]. Heavy industrial chemicals need to be approved and regulated. Further, the chemical handlers must be provided with a material safety data sheet. International bodies such as the Codex Alimentarius and the United States Food and Drug Administration have introduced standards to alleviate the chances of food contamination through cleaning agents. These standards are voluntary but relevant, especially if companies want to trade internationally. Some compounds when not properly diluted cause adverse human health effects such as chronic dermatitis upon direct contact or prolonged use [20]. This could be poisonous when introduced directly into food [21,22,23]. An array of products has been endorsed and others discontinued due to their toxic effects on humans and the potential margin of damage in the event of food contamination. Historically, peroxides and most ammonium products are universally accepted within the specified scope of use and safety thresholds. Therefore, research must be conducted to determine the safety threshold of most cleaning agents in the food industry.

### 2.3. Food Additives

**Figure 2:**
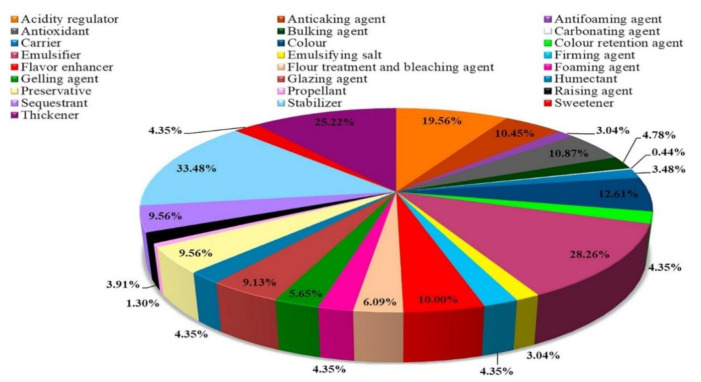
Percentual relationship of each class of the food additives used in food industries [32]. Copyrights Elsevier.

Advancement in research in the food industry has been rapid and certain technologies have been introduced to counter food perishability and to reduce the amount of food wasted due to microbial degradation. However, these technologies need to be introduced and used judiciously because of their potential to cause food-related illnesses [24,25,26]. Food additives represent some of the innovations introduced in the food industry to alleviate waste and prolong the shelf life of foodstuffs [27,28,29]. It is estimated that each person may consume close to 3.6 to 4.5 kg of food additives on average per annum [30]. Food additives are described as “substances of natural or synthetic origin, which are added to foods to serve a technological or sensory function” [31]. The definition is further expanded and described by the Codex Alimentarius as any substance that is not normally consumed as a food, but it is used as a typical ingredient of the food to serve the purpose of adding nutritive value [30,32]. Furthermore, the addition of the substance will directly or indirectly be a component of the food. As shown in Figure 2, the synthetic materials in food can be categorized into different sub-categories according to their function in the food i.e., colorants, emulsifying salts, flavor enhancers, acids, packaging gases, sweeteners, thickeners, etc. Moreover, food additives can further be classified as natural, synthetic natural, modified, and artificial additives [25,28,32,33].

The impact of food additives has been far-reaching, hence the World Health Organization (WHO) and other international forums promoting their inclusion in risk and safety activities despite the scientific uncertainty of some contaminants [31]. There are debates amongst the scientific community and various consumer interest groups regarding toxicologic studies and toxic levels. Residues such as bisphenol A, mineral oil aromatic hydrocarbons, and synthetic amorphous silica are not well studied and there seems to be uncertainty regarding exposure assessments and potential health effects [34]. At the consumer level, the influx of illegal food additives is perceived differently. Further, consumers’ usage of food additives may be influenced by the market anchor price [25,35,36]. Essentially, their decision to use or not use additives is determined by intuition and experience due to their lack of expertise in food safety [34].

#### Preservatives

Food additives have several functions. Depending on the type, some may serve as chemical preservatives [24,30]. The transformation in consumer needs and the food industry in recent years has led to non-conventional methods of ensuring high yields with minimal cost to producers. Consumers have gravitated towards high-energy food with a distinct flavor and reduced preparation time [30,36]. The food industry stands to lose production if the food has a shorter shelf-life; therefore, technologies are used to curb the effect of food spoilage on the economy and company profits [37]. Preservatives are compounds that promote the reduction or prevention of microbial growth in various foods and products [24,25]. Moreover, preservatives can be synthetic or natural substances that are usually used in low concentrations to inhibit the growth of bacteria [38]. Two classes can therefore be distinguished: class I, which includes natural preservatives, and class II, which contains chemical or synthetic preservatives. Examples of chemical preservatives include sorbate, benzoate, and nitrates [24]. These synthetic preservatives pose a public health hazard in uncontrolled doses. Effects such as headaches, palpitations, allergies, vomiting, and skin rashes have been documented in past studies [24,28]. In other toxicologic studies, synthetic preservatives have been reported to have genotoxic and carcinogenic effects [30]. A study in China between the years 2006 and 2015 revealed that 34.36% of all food safety incidents (N = 253,617) were a result of the illegal use of food additives.

### 2.4. Food Packaging

Food packaging serves various functions in the food industry. The benefits extend from branding and advertising, barrier protection, protection from external elements, and to some extent, food preservation [16,39,40,41,42]. Generally, food packaging encompasses a lot of processes whereby different food additives are used and blended with polymers to make the resultant material more durable [5,43]. In food packaging, chemicals are intentionally applied in the manufacturing process and other materials are constantly in contact with food along the food production chain [44]. It is well documented that food packaging processes can introduce toxic contaminants in food, thus causing public health problems [45,46,47]. Research shows that food contact chemicals can have harmful effects on humans. Therefore, stringent measures need to be devised to address this challenge. Furthermore, these food contact chemicals come in various ways, namely: food packaging, food storage containers, kitchen utensils, and food processing equipment [39,48,49]. The process of transferring and partitioning chemical compounds from food packaging into food through adsorption or diffusion is known as migration [45,50,51]. Essentially, migrants from packaging have the potential to cause adverse effects on human health.

Food contact materials (FMCs) can release migrating substances. FMCs are reportedly the leading source of chemical contamination in food and significantly contribute to chronic chemical exposure [44]. Migration of contaminants into foodstuffs depends on factors such as the compositions of the material, package size, temperature, storage time, the nature of the food, and how it is exposed [45,52]. As a result, food packaging is highly regulated internationally due to a vast range of literature on the carcinogenic effects of some of the chemical compounds used as ink and food protective membranes [39,53]. In some instances, incorrect packaging methods, incorrect storage, and handling cause structural deficiencies in the packaging thus leading to bloating of canned foods and sealants leaking into the food product. Moreover, corrosion through oxidation can alter the structural integrity of metallic containers, thus posing a health hazard. For this reason, metallic containers are coated with epoxy resin varnishes to curb corrosion [5]. Typical migrants from contact materials are chemicals such as benzene, especially in the flavored beverage industry. This chemical is used extensively in the manufacture of plastics and pesticides and it is known to have carcinogenic properties. Another chemical compound used in the food industry is bisphenol A (BPA) which is used in the lining of some food and beverages to extend their shelf life [48]. It usually serves as a plasticizer in polymers like polyvinyl chloride (PVC) [39,54]. Table 1 shows the types of packaging used in a variety of foodstuffs and how contamination is introduced.

Food products such as sugar, maize, flour, rice, and most cereals are usually packaged in paper or board materials. For this reason, there are possibilities of chemical contamination by exposure to printing ink or, in extreme cases, absorption of moisture and chemical leaks when stored directly on the floor [44]. Polymers are widely used in various applications in the food industry. Extensive research has progressed their usefulness in material design and general safety. Notably, polymers are used in food packaging and the most common are poly-ethylene, polyvinyl chloride, polystyrene, polycarbonate, and polyethylene terephthalate [5]. Phenolic endocrine-disrupting chemicals in food through food packaging have been largely reported in canned food, disposable plastic bottles, polyethylene terephthalate, etc. This happens by the migration of chemicals from food packaging over time after being exposed to conditions such as heat and normal tear and wear [55,56].

Researchers in one study asserted that eco-friendly packaging might have passed tests in environmental sustainability and suitability; however, they warned that usually the human risk factor tests are not rigorously documented for the same packaging material [46]. Basically, in a life cycle test, a material can pass the first stage only to cause adverse effects in another phase. A human being is routinely exposed to harmful chemicals from a variety of sources such as food [17]. This calls for more toxicological studies to be conducted with mammalian subjects to measure the risk for individual chemicals. However, effective toxicological studies need to have well defined and understood contaminants. This ensures that appropriate regulatory reviews are conducted to optimize testing for chemical migration levels [47,57]. Therefore, the identification of all chemicals inherent in the food and packaging material is critical. According to Karmaus and co-workers [47], this process is completed through the evaluation of technical datasheets, supplier information, material safety data sheets, and compliance letters.

**Table 1:** Summary of selected food packaging and how contamination is introduced.

Type of Food	Packaging Type	Contamination Pathways	References
Rice	paperboard	Adhesives, coatings, inks	[47]
Maize	paper	Migration, moisture, inks	[58]
Meat, Fish	Polystyrene, corrugated fibreboard	Moisture in humid areas	[58,59]
Sugar	Paper, paperboard	Absorption of moisture and chemicals.	[58]
Raw and processed fruits/ vegetables	Polystyrene, metals, vegetable parchment paper, moulded pulp packaging	Moisture absorption and migration	[42,58,59]
Dairy	Polystyrene Plastics, metals, folding cartons	Migration and leaching chemicals	[42,59]
Bakery	Polystyrene, greaseproof paper	Moisture absorption	[58,59]
Beverages	Metals, composite cans, foil wraps	Migration of Bisphenol A Blackening, corrosion, bulging, tin dissolution, leaching coatings	[42,58,60]

#### Innovations in Food Packaging

The 20th century provided technological advancement regarding the use of smart packaging and active packaging [61]. According to Yucel (2016), food packaging accounts for half of all packaging [62]. Contributions in the literature by various researchers shows that the food industry has increased the use of flexible and plastic packaging such as low-cost polyester, alcohol ethylene polymers, and polypropylene [41,45,62,63]. This rise in technological innovations provides opportunities for increased food safety, economic growth, and reduced loss of products [64]. This increase in the use of technology is also influenced by trends in food demand. Mania et al. (2018), stated that the “transregional and transnational long-distance” movement of food requires innovation to keep the food appealing to the consumers [40]. However, the use of new materials and technology can introduce foreign materials in unacceptable quantities. Active, green, and intelligent packaging can introduce contaminants through chemical migration. Therefore, all food products must be properly labelled in consideration of the material used in packaging and the potentially toxic ingredients declared by legislation. Essentially, all additives in food packaging must be inspected and approved by an inspection authority or regulatory body.

Industrialization in the food industry has called for increased demand in the use of plastics in packaging. Plastics can be described as materials that are of synthetic nature or natural polymers [45]. Furthermore, they can be modified and manipulated using factors such as heat and pressure. Examples of plastics include bowls, foils, bags, and bottles [44,52]. Plastic materials are highly diverse and can be recycled. It is important to note that during recycling, polymers are partially degraded due to the breaking of intracellular bonds, leading to a change in their mechanical structure and appearance. During the recycling process, other non-intended chemicals may be introduced on the food contact surface leading to cumulative toxic chemical effects. The chemicals in the plastic can migrate in several ways. Firstly, by direct contact of the food with the inner layer of the wrappings. Secondly, the chemical substance can indirectly pass one layer before being in contact with the food. Thirdly, it is possible that there may not be any physical contact. The space in food containers can be a mechanism for migration, especially with volatile substances [65]. For this reason, it is vital to conduct exposure assessments on all chemicals that can be potentially consumed as per the WHO recommendation [66]. Even though studies are constantly conducted to determine the toxicity of chemicals, they are usually executed using food simulants and focusing on specific compounds, not the final product. Fast screening of compounds from plastic packaging can be done; however, there are still challenges in determining the toxicity of the final food product.

## 3. Environmental Chemical Contamination

Environmental pollution is a widely documented burden in the ecosystem [5]. More recently, the effects of such pollution have been reported to affect the food production chain. Amongst the major contaminants in the food production system, chemicals have been classified as a significant contributor to food contamination. The lack of management systems or weakened public health interventions, especially in high-density industrial areas of developing nations, has historically been linked with the contamination of food by chemicals [5,67]. This supports the hypothesis that there is indeed a relationship between environmental contamination and public health despite the association being poorly documented and reported. In many settings, it is difficult to quantify the extent of the contamination due to diverse contagions and lack of advanced expertise.

There are multiple pathways to the contamination of food. The most common mediums contaminated by chemicals affecting the food industry are the water, soil, and direct contamination due to anthropogenic activities. Inadequate hygiene and poor sanitation are perceived to be among the leading causes of pollution and the manifestation of diseases globally [67]. Water pollution particularly has a significant impact as it is an essential part of any food. Moreover, the contamination of groundwater sources and changes in the chemical composition of water alters biotic and aquatic systems. There are two key water contamination types; firstly, change in waters’ physical properties, as well as the amount of matter moved by the aquatic system. Secondly, change in the chemical composition of the aquatic body [67]. Contamination can have a disastrous outcome in the food chain and, similarly, the food production chain can have a detrimental effect on water pollution. In agricultural environments, water runoff and chemical leachate can pollute water sources. Furthermore, manufacturing plants including mining operations can emit chemical pollutants enough to contaminate raw food products.

### 3.1. Persistent Organic Pollutants

Persistent Organic Pollutants (POPs) continue to be a threat to the environment due to continued emissions. The Stockholm Convention on POPs in the year 2001 restricted the usage of POPs; however, it was only in 2004 that the resolution was put in effect [68]. Long after the adoption by the conference committee, the continued exposure to POPs can be attributed to heavy industrial activities which include the waste management sector and other industries that use additives and pesticides [68,69]. Persistent organic pollutants are carbon-based and can be in vapor form or as adsorbed by atmospheric particles [70,71]. The commonly known POPs are dioxins, dibenzofurans, organochlorine pesticides (OCPs), polycyclic aromatic hydrocarbons (PAHs), and PCBs [70,72]. Additionally, these compounds have been reported to have a long-lasting effect on the environment due to their non-degradable nature [17,70]. In Africa, the biggest contributor to POPs is pesticides. This is observed chiefly in countries where food production and trade contributes significantly to the gross domestic product (GDP). Furthermore, Ghana is reported to be one of the top pesticide users. In a review study in Ghana, dichlorodiphenyltrichloroethanes (DDTs) were found to be high risk in the studied food groups. However, the decline in some of the results is assumed to be a result of the Stockholm convention declaration [69]. The researchers further noted that people who are not exposed to POPs in their normal work environments are exposed through their dietary intake of animal products. Their exposure can be worsened by the ingestion of fruits and vegetables contaminated by pesticides.

#### 3.1.1. Polychlorinated Biphenyls (PCBs)

Polychlorinated biphenyls (PCBs) are a group of manufactured synthetic chemicals falling under the umbrella of POPs. They were first produced in large quantities in the 1940s until the late 1970s; however, the first synthesis was in the 1880s [73]. These chemicals have clear color and can be presented as solids or liquids. Historically, PCBs were used as lubricants, plasticizers, and insulating oils for capacitors and transformers [74]. Furthermore, they are classified as persistent organic compounds due to their long-lasting effects on the environment. PCBs are known for their stable properties, thus being able to withstand temperature extremes and pressure. Generally, biphenyls can be formed through chemical manipulation of various organic mixtures such as plastics and harvest protection chemicals [67,75]. Literature suggests that the common route of exposure to PBCs is through the ingestion of contaminated foodstuffs [75]. Moreover, the highest levels of contamination have been noted in fish, meat, eggs, and dairy products [74]. This is attributed to the high daily consumption of these foodstuffs. The ability of PCBs to infiltrate the food chain has been attributed to the fact that they are lipophilic, persistent and they can accumulate in the environment for prolonged periods, notwithstanding that animals with a long lifespan have been reported to accumulate PCBs in their fatty tissues at high levels.

Earlier studies in Japan and Taiwan have reported food poisoning outbreaks as a result of PCBs in 1968 and 1979 respectively [73]. In these studies, the contaminated foodstuff was rice, which is a staple food in these countries. Affected people exhibited symptoms such as pigmentation of the skin, numbness in the limbs, acne-like eruptions, and abnormal discharge from the eyes [73]. Other studies conducted in Lanzhou, China showed increased levels of PCBs in food compared to non-industrialized areas. In this study, the highest concentrations were discovered in aquatic products (0.31 ± 0.30 ng/g). Furthermore, eggs and meat had the next-highest concentrations (0.08 ± 00.9 ng/g and 0.06 ± 0.05 ng/g). The results showed high levels of exposure especially regarding the dietary intake of staple foodstuffs [74]. A review paper by African researchers supported the hypothesis that generally urban development centers and industries account for the largest burden of PCB exposure in industrialized communities [76].

#### 3.1.2. Pesticides

A pesticide is a common name for all plant growth regulators, fungicides, herbicides, insecticides, rodenticides, molluscicides, and nematicides [77,78]. Pesticides are widely used globally due to their benefits in controlling the manifestation of pests. They can be applied throughout the food production chain i.e., farm, production, storage, transportation, distribution, processing, and at the consumer level [7,71,78,79,80]. They are essentially chemicals used to mitigate against pests that cause plant diseases. They are known to affect “target as well as non-target species” [67,81]. The growth of the agricultural sector has also increased the usage of pesticides over the years. The first-generation pesticides were manufactured in the 1860s and later discontinued due to their toxic effect. In the 1870s, synthetic organic compounds were introduced [77]. However, it was not until the 1940s that pesticides were manufactured and used extensively. In addition, the rise of industrial production in both developed and under-developed nations increased the usage of pesticides in their forests and crop fields. The general long-range transportation of pesticides makes them pollutants that transcend local, regional, and national boundaries [82,83]. Pesticide poisoning has a detrimental effect on human beings. As reported by Bhalla and colleagues, 250,000–370,000 people die every year due to the direct or indirect ingestion of pesticides [7]. Moreover, between the years 2010–2014, Japan was reported to use pesticides more than any country. This demonstrates a heavy reliance on pesticides in some countries due to a lack of cost-effective alternatives.

**Figure 3:**
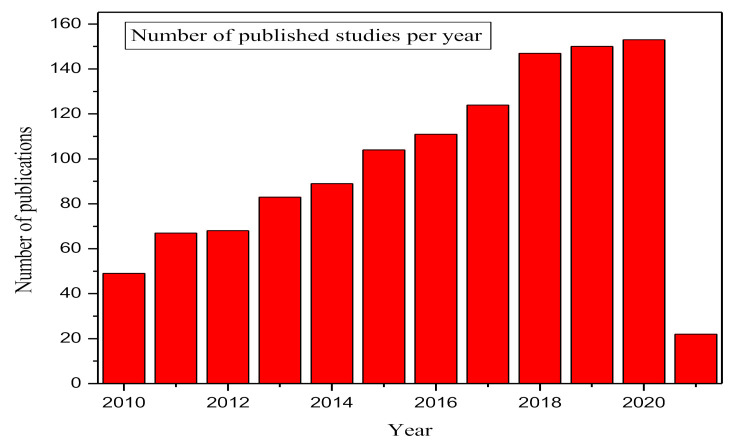
Pesticide contamination in food and the number of outputs per year. Data analysis was completed using the Web of Science databases on 4 March 2021 by the authors.

Figure 3 shows that there has been a steady increase in the study of the contamination caused by pesticides in food. Figure 3 shows the number of published studies from 2010 to March 2021 retrieved from the Web of Science core collection database (SCI-EXPANDED, SSCI, A&HCI, CPCI-S, CPCI-SSH, BKCI-S, BKCI-SSH, ESCI). The Web of Science core collection database is known for its robust scientific authority and frequent use in bibliometric analysis by various researchers [84]. The closest matching search terms selected to identify publications by topic were “Pesticide food contamination” and “Health effects” or “contamination”. The search items produced 1167 results over a wide variety of sources such as peer-reviewed articles, book chapters, review papers, etc.

The most-studied pesticides are organophosphorus pesticides (Table 2). The contamination from these pesticides can be removed by using microorganisms in the process of bacterial degradation [85,86]. Past studies as reported by researchers have shown that lactic acid bacteria such as *Lactobacillus bulgaricus* and *Streptococcus thermophilus* have the ability to degrade pesticides [85,86]. Moreover, enzymes such as phosphodiesterases, methyl parathion hydrolases, and organophosphorus acid anhydrolases can degrade organophosphorus pesticides. Other studies [87] have reported *Saccharomyces cerevisiae* yeast during fermentation as a potent option in the removal or reduction of some pesticides in food. Therefore, to control the risk of pesticide exposure, risk assessment models need to be applied in food production industries and retailers to assess the level of pesticide residues [87,88].

According to Kumar, Chand, and Shah (2018), approximately 80% of all pesticides globally are produced in developed nations annually [77]. Moreover, farming activities account for approximately 70–80% of pesticide use. Their application on vegetables alone in developed countries is estimated to be around 25% [78]. It has been cited that only 0.1% of pesticides actually reach the intended pest, and the remaining 99.9% proliferates in the surrounding environments i.e., food, water, air, etc. [67]. Essentially, pesticides are transported in environments through various ways such as water runoff, spreading through the vapor, leaching into substances, and shifting from different places through osmosis under the influence of air circulation [77,78].

**Figure 4:**
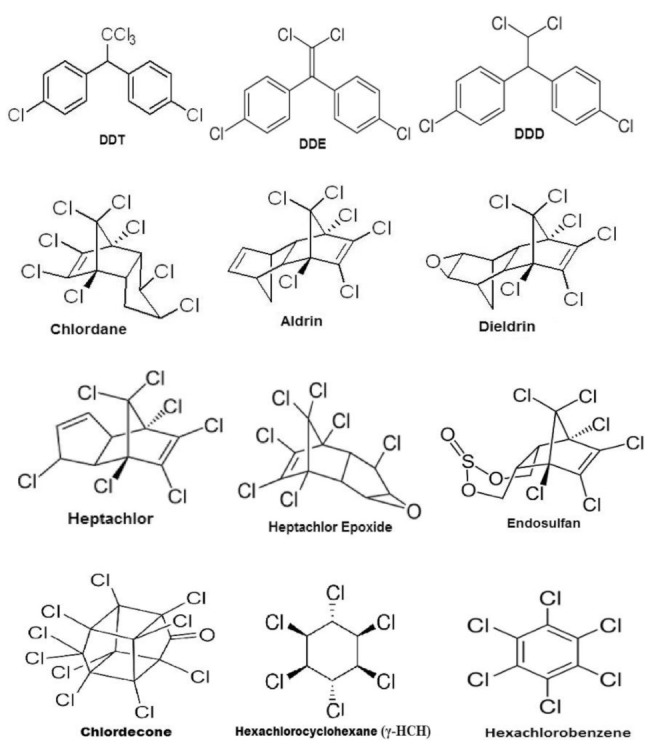
Structure of some of the organochlorine compounds [82]. Copyrights Elsevier.

There are about 1400 known pesticides [48]. Some pesticides such as DDT have long been banned in some countries, however, bioaccumulation is still detected in some streams due to the lasting effects of the chemical compounds [91]. Therefore, rigorous monitoring standards and regulations need to be adhered to across national borders [82]. In risk assessment studies, it has been found that the cocktail effect of chemicals is even more dangerous due to multiple exposure pathways [90]. Essentially, the additive effect of one compound is heightened when it is combined with another. DDT can last in the soil for years until it enters the food chain through adsorption and eventually contaminates the food. Other chemical compounds such as organochlorine pesticides (OCPs) have been widely studied in recent years due to their agricultural use [82]. These OCPs are known for their chemical qualities (Figure 4). Over the years, they have been reported to be almost everywhere, persistent, hydrophobic, and resistant to degradation. Organochlorine pesticides are chemically stable and semi-volatile, which implies that they last longer in environments and can be transported easily in the atmosphere through the wind, and they are known to be lipophilic. This trait makes them attach easily to animal and human fatty tissues. OCPs are majorly found in fatty foods due to their fat solubility, e.g., fish, meat, and dairy products. African countries such as Togo, Nigeria, and Ghana still report figures higher than the maximum permissible limits due to their extensive use [82].

#### 3.1.3. Pesticides and Food Safety

Daily consumption of food comes with potential daily exposure to pesticides. Most countries have pre-determined maximum permissible limits as recommended by FAO/WHO/Codex Alimentarius. In Europe, the European Food Safety Authority (EFSA) conducts independent research on risk assessments and further advice authorities on permissible levels. Essentially, the EFSA functions as a gatekeeper to the European Commission regarding the approval of new substances and setting new maximum residue levels after completing their rigorous peer review and due diligence on potentially harmful substances [92,93]. In cases where pesticide residues exceed the maximum residue level, the exposure must be compared with the acceptable daily intake (ADI) and/or the Acute Reference Dose (ARfD) to assess the risk for the consumer [78]. Researchers define the ADI as “the number of pesticides in mg/kg to which humans can be exposed to daily through ingestion during a lifetime without appreciating risks to the health on the bases of all known facts at the time of evaluation” [78,94]. On the other hand, the ARfD is established for the general population based on children and infants, including women who are considered to be of childbearing age. Essentially, it is the exposure level at which harmful effects are likely to occur in the most sensitive individuals in a population during a single day exposure, within 24 h. Member countries affiliated with the WHO have established threshold limits for food contaminants to comply with recommendations as defined in the Codex Alimentarius. Table 3 shows the maximum allowable levels of pesticide residue that may be present in food according to the Codex Alimentarius standards.

## 4. The Use and Effects of Antibiotics in the Food Industry

The history of the use of antibiotics in agriculture can be traced as far as 1935 by German pharmaceutical manufacturers [96]. There have been significant strides in the technology relating to the usage and rapid manufacturing processes in food safety industries. Antibiotics can be viewed as chemical compounds used to inhibit the growth of bacteria [97,98]. These antibiotics can be produced naturally or synthetically in laboratory conditions [27,99,100]. They have been used extensively over the years in medical applications due to the high demand for medical drugs for human and animal health [1,48,101]. This demand has seen an increased risk of exposure of humans to various antibiotics, both as a by-product or by direct ingestion. In particular, the risk can be a result of a prolonged intake of contaminated food products. Crops and meat products serve as examples of typical foods that may be exposed to antibiotics. In animal farming, antibiotics are hazardous to humans when no quality control measures are observed, thus resulting in humans consuming them in large and unsafe quantities [99,102]. According to Bacanli and Barasan (2019), 80% of animals used in food production are currently treated with veterinary drugs or will be throughout their lifetime [99]. Therefore, it is vital for the food industry to constantly analyze the methods and standards used in the administration of antibiotics to avoid food-borne drug residues. These food-borne drug residues can cause both chronic and acute health effects. Table 4 summarizes some of the antibiotics in food including their human health effects.

**Figure 5:**
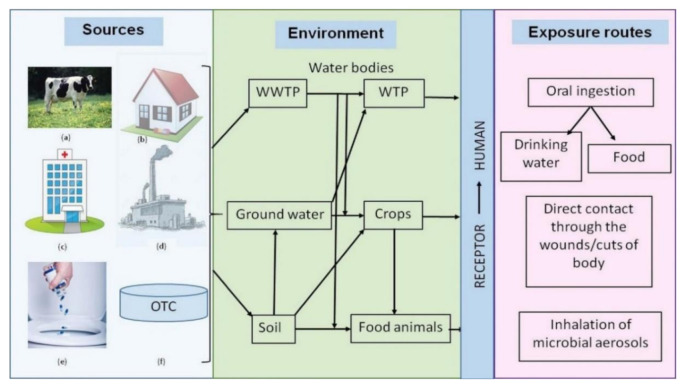
Sources of antibiotic usage, its spread, and transfer of resistance genes to humans. The excessive usage of antibiotics as growth stimulants in livestock and other food animals can contaminate water sources when animal excreta is washed off with water into the environment (**a**). The contamination of sewage treatment plants can be a result of excessive human usage of antibiotics (**b**). Hospitals and pharmaceutical industries contribute significantly to wastewater treatment plants’ pollution by antibiotics when they are illegally let into sewage systems (**c**,**d**). Improper disposal of antibiotic pills and unprescribed over-the-counter antibiotics can contaminate wastewater treatment plants (**e**,**f**) [104]. Copyrights Elsevier.

**Figure 6:**
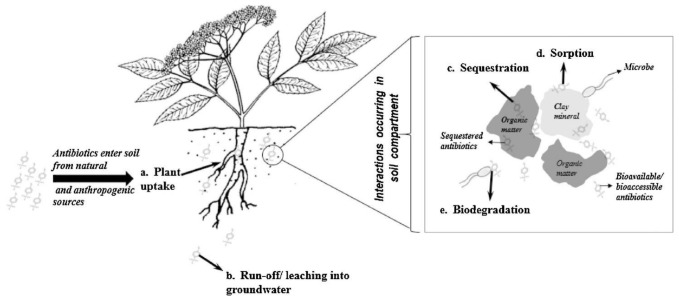
Fate of Veterinary antibiotics [114]. Copyrights Elsevier.

The main use of antibiotics in food animals as suggested by various sources of literature include the prevention of diseases and promoting growth [99,102,103,104]. Antibiotics can be administered in various ways such as orally or parenterally. Thus, incorrect handling and application can be a public health disaster. The residues in foodstuffs can cause adverse effects on human health. Additionally, contamination occurs through the feed, drinking water, food processing equipment, and processes [104]. The excreted antibiotic dose (30–80%) provided to food animals can enter the environment through manure or fertilizer which can ultimately be used as a plant nutrient or animal feed, thus completing the vicious cycle in the food chain [104,105]. Various ethical and environmental issues regarding the use of antibiotics have been debated in recent years. Chief amongst the ethical issues is animal health regarding the procedures for administering the antibiotics. Inappropriate therapy can further cause antibiotic resistance in both animals and farmworkers [106]. Figure 5 and Figure 6 illustrate the exposure routes of antibiotics, transfer of resistance genes to humans, and the fate of veterinary antibiotics.

**Table 4:** Summary of the classification of antibiotics and human health effects.

Pesticide Name	Class	Documented Health Effects	References
Oxytetracycline	Tetracyclines	Poor teeth development in young children and stained dental enamel, loss of appetite, diarrhea	[100,107,108]
Pleuromutilin	Timulin	Suspected metabolic instability, hepatotoxicity, concerns around cardiac safety, lack of sufficient oral bioavailability, gastrointestinal side effects	[109,110,111]
Ampicillin	Aminopenicillins	Angioedema. It can cause stomach cramps, diarrhea, dizziness, and rashes nausea. Overdose can cause confusion, blackouts, and renal failure	[108,110,112]
Erythromycin	Macrolides	Abdominal pain, cramping, Nausea, vomiting, and diarrhea	[108,113]
Sulphonamides	Sulphonamides	Pruritic rashes, gastrointestinal distress, hematologic abnormalities, and fever	[98,108]
Difloxacin	Quinolones	May cause central nervous system toxicity, especially in animals with renal failure. May cause some nausea, vomiting, and diarrhea at high doses	[98,109]
Enrofloxacin	Quinolones	Central nervous system stimulation may lead to restlessness, tremors, confusion, and hallucinations	[98,109]
Flumequine	Quinolones	Adverse reactions were observed, including vomiting	[109]
Nalidixic acid	Quinolones	Convulsions, increased intracranial pressure, and toxic psychosis	[109]
Oxolinic acid	Quinolones	Nervous excitation, stereotyped behavior, and insomnia	[98,109]
Trimethoprim	Potentiator	Pruritic rashes, gastrointestinal distress, hematologic abnormalities, and fever	[98,110]

## 5. Heavy Metals

Various metals are essential for living organisms; however, at an excessive concentration, they can be detrimental to human health [115,116]. Literature shows that certain elements are studied more than others according to the geographical location [115]. According to Antoniadis et al. (2019) metallic elements such as nickel, copper, iron, aluminium, manganese, chromium, cadmium, lead, argon, zinc, and arsenic have received more attention in literature with regards to human health impacts [115]. The researchers assert that this could be attributed to their significance in the human food chain, especially concerning the allowable maximum daily dietary intake. The researchers also mention an interesting narrative about crop production areas and their locality. Essentially, the more an area is used for agricultural produce, the more industrial parks grow around it. In turn, the industrial activities will contaminate the food through the water table and the air, thus affecting human health. This is supported by Al-Othman et al. (2016) and Rai et al. (2019) who revealed the long-term degradation of the quality of the environment and adverse human health effects as a result of industrialization [117,118]. This provides conclusive evidence that plants grown in polluted environments can be a vehicle for contaminants to be introduced into the food chain. Therefore, Organizations such as the European Chemical Agency came with strategies to protect human health by heavily regulation the use of chemicals. This ensures control through the identification of hazardous substances and monitoring [119].

Heavy metals are metallic chemical elements that may be toxic and poisonous when untreated. They are known to bio-accumulate and persist in the environment. Moreover, they can enter the human body through direct contact or ingestion of contaminated foodstuffs [33]. Fish contaminated with methyl mercury from industrial effluents have been reported in the past where neurological symptoms were seen in patients who consumed the fish [67]. Sustenance is essential for human life, therefore exposure to factors adverse to human health through foodstuff is of high importance. The scarcity of food in developing countries increases the risk of exposure to harmful untreated metals. In communities relying on aquatic food for sustenance, the risks of digestive ailments are high especially when exposed to metals such as copper (Cu) which is known for causing digestive discomfort in susceptible individuals. Heavy metals are majorly introduced in the human body through two common pathways. Firstly, the inhalation of contaminated air. Secondly, the ingestion of contaminated water and food plants grown in the regions where the soil is contaminated [117,120]. In addition, in studies assessing the presence of toxic metals in wheat crops grown on selected soils irrigated by different water, researchers discouraged the propagation of wheat plants near highly industrialized areas due to the heavy metals’ tendency to accumulate in the aerial parts of the plants, thus explaining phytotoxic properties of the metallic compounds [117,120].

**Figure 7:**
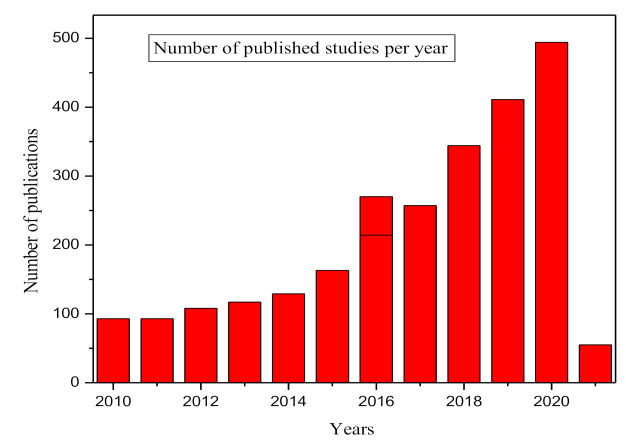
Heavy metal contamination in food and the number of outputs per year. Data analysis was completed using the Web of Science database on 04 March 2021 by the authors.

Figure 7 shows that there has been a consistent gradual increase in the study of heavy metals in food in terms of the number of published studies since the year 2010. The Graph shows the number of published studies from 2010 to 04 March 2021 as recorded by the Web of Science core collection database (SCI-EXPANDED, SSCI, A&HCI, CPCI-S, CPCI-SSH, BKCI-S, BKCI-SSH, ESCI) using the search string ‘Heavy metal contamination in food’.

### Pathway of Heavy Metals

Heavy metals are transferred along the plant pathway in various ways. The most common is through soil spores to plants in ionic forms [118,121]. The capillarity of plants enhances the translocation of heavy metals to the rest of the plant. Therefore, the concentration of heavy metals in plants may vary according to the different parts of the crop plants i.e., roots, leaves, and fruits. The roots of the plants play a vital role in the rate of metal absorption and translocation to the rest of the plant. Different crop species have membranes that may be more adapted to rapid absorption than others [117,122,123,124]. A study was conducted in Nigeria whereby a Transfer Factor (TF) for four heavy metals was analyzed in five different vegetables that grew near the discharged wastewater sites. The most prominent result in the study was the high TF of cadmium (Cd) in the Irish potato (TF of 2.88), attributed to the concentration of metals in the soil. The overall TF of accumulated cadmium in the overall samples was 6.52 × 10^−5^–2.88, while Lead (Pb) had the lowest accumulation with a TF of 0.23–0.34. Further analysis showed that chromium and cadmium were above the maximum permissible limits for vegetable consumption [125].

One important factor to consider is the significance of crops such as wheat and maize in developing nations such as South Africa. These crops form the country’s staple foods or national diet. They are distributed and sold to communities that may already be immuno-compromised or already under community or household level of food insecurity. The populations likely to be affected include schools under national school feeding and nutrition programs, hospitals, prisons, and indigents receiving food parcels. This is because the above-mentioned population groups do not have a range of options regarding what they can consume. In Southern Africa, food products such as maize and peanuts are considered primary staples [126] and contribute significantly to the economy in the southern region. The researchers state that quality crops are exported while the poor crops are left for local consumption. This can be attributed to the high demand and standards of the developed nations. The typical climatic conditions (warm and humid) in the southern region are conducive for the manifestation of toxin-producing fungi and possibly the disintegration of certain polymers used in food packaging.

Maize in South Africa is a staple that is usually consumed fresh or processed, cooked, or fermented. Another factor is the contamination of vegetables by metals in peri-urban or rural agricultural holdings. Therefore, along the production chain, maize could be contaminated by metals in the soil. By implication, subsistence farmers especially in the rural parts of the country may be exposed to adverse effects of contaminated food. Table 5 shows the maximum allowable limits for heavy metals allowed in foodstuffs according to the Codex Alimentarius International standards for Food. The higher chemical concentration exposure by agrochemicals and trace elements can be attributed to the location of the heavy industries which are usually located outside the city centers as per town planning schemes [90].

## 6. Food Safety Laws

There has been an increase in the international trade of food globally. Developed nations have come up with innovative and stringent measures to protect against food contamination through food safety management systems that aim to improve traceability and transparency from partner countries [128,129,130]. Moreover, it is reported that suppliers along the food production chain are increasingly showing their importance in food safety and the application of international and regional laws. This is due to incidents of failing food safety management systems threatening food safety in recent times across the world. The WHO and the Food and Agriculture Organization (FAO) have joined forces in terms of defining and setting international standards for food safety regarding safe exposure levels and educational programs. The regulatory measures and laws by these organizations are meant to promote quality and safe food supply to participating member states across the world [130]. Furthermore, the interventions agreed by these organizations gave rise to committees on food additives established in 1965 and the expert committee on pesticide residues [7]. The FAO/WHO collaboration also has the Codex Alimentarius initiative established in 1963 which aims to supervise and facilitate the refinement of definitions and requirements for food to make transboundary standards consistent. The Codex Alimentarius has committees mandated to deliver specific standards in selected food commodities. Notably the codex committee on contaminants in food (CCCF) [49], codex committee on pesticides residues (CCPR) [131], and the codex committee on residues of veterinary drugs in food (CCRVDF) [132]. These committees are set up to establish standards and to develop the code of practice for contaminants in food. This includes methods of sampling, analysis, and maximum exposure limits [93].

Food safety incident rates gave rise to the promulgation of the ISO 22000 international standard for establishing food safety management standards. This standard has seen several revisions from ISO 22000:2005 to the current ISO 22000:2018. It has come with opportunities and challenges in its interpretation and application. Essentially, this standard aims to assist organizations with optimal allocation of resources, improve communication internally and externally, document organizational performance, build consumer trust and monitor management performance [7,128]. Over the years, most organizations have used a common hazard analysis critical control point system (HACCP); however, it has come under scrutiny in recent years for its application in small- and medium-sized enterprises [128]. The main criticism was regarding organizations’ over-reliance on applying critical control points in operation without the adequate training the ISO 220000 standard recommends. Ultimately, a tiered approach of different standards is recommended to effectively deal with dynamic food safety concerns.

## 7. Regulatory Strategies

Organizations are encouraged to adopt self-regulatory approaches where they can use external approval from inspection authorities to monitor the food safety compliance levels within the organization. In the United Kingdom, the government compels companies to design a set of policies and laws and then an external company audits the company against the set standards [129]. This process is done through hazard analysis by identifying risk factors inherent to the business and ultimately the consumers. The other regulatory strategy is enforced by Environmental Health Practitioners (EHPs) who are usually appointed by local authorities. They play an advisory and educational role within the food industry. Other functions include issuing statutory notices, premises closure, and prosecution [129]. In a study in the UK, money was found to be an important element to prompt small–medium enterprises to comply with food safety standards. Finances need to be considered when designing food safety protocols and their impact. Companies can be reluctant to spend money on food safety measures due to a lack of commitment to food safety. As a consequence, consumers will be negatively affected.

The regulation of the food industry and consumer behavior in food safety matters is a challenge in South Africa. In the food retail sector, there have been numerous reports on bacterial infections and pesticide residues on foodstuffs distributed for sale [133]. Factors leading to food contamination include unhygienic food preparation and, in some cases, sheer negligence by both the retail sector and consumers. The food retail sector is the leading cause of food safety incidents due to the vast supply chain and the sector being the last stage before the food reaches the consumers [133]. South Africa is an emerging economy and the food industry has been reported to have contributed 9% to the overall gross domestic product in the year 2016 [133]. This can be attributed to the dominance of supermarkets and farms that supply them.

Southern Africa and the rest of Africa largely depend on agriculture for subsistence [68]. In South Africa, the food industry is regulated by three departments, namely: the Department of Trade and Industry (DTI), The National Department of Health (NDoH), and the Department of Agriculture, Forestry and Fisheries (DAFF). These departments are further entrusted to oversee certain sets of legislation according to expertise and designation. The Consumer Protection Act (Act 68 of 2008) is enforced by DTI and it supersedes all food-related legislation in South Africa. The second in the hierarchy of powers is the NDoH which is mandated to enforce the National Health Act, related amendments (Act 61 of 2003), and the Foodstuffs, Cosmetics and Disinfectants Act (Act 54 of 1972). The Department of Agriculture, Fisheries, and Forestry is entrusted with the enforcement of the Agricultural Product Standards Act (Act 119 of 1990). All these pieces of legislation combined form the core of the South African food control system.

## 8. Conclusions 

The study of chemical toxicity in the food industry is fundamental and needs to be supported by rigorous toxicological studies. This will improve the quality of food products offered by the food industry and will ultimately benefit consumers. Heavy metals, antibiotics and POP contamination can cause adverse human health effects and thus needs regulation through adequate legislative interventions and proper monitoring standards supported by sound scientific data. The already existing interventions such as the bioremediation of pollutants are effective. However, more research needs to be conducted on the sustainability and financial impact of these solutions as a control strategy in the food industry. This review revealed a plethora of studies that could be undertaken to further narrow the toxicological effects of chemicals on food sources.

Further research might explore how toxic chemicals in food are transferred from farms to consumers in developing countries. This kind of toxicological study might compare the toxicological effects by region and even the proximity of exposure to food sources and the environmental drivers of food contamination. Threshold limits need to be developed for various chemicals at low concentrations. An in-depth study could further explore the food safety management systems in place at the national level of food control including the relationships with stakeholders and consumers. This paper highlighted that most studies on heavy metal exposure were conducted in Asian countries such as China and Japan, notwithstanding the contribution of the European Union through the Registration, Evaluation, Authorization and Restriction of Chemicals (REACH). Significant work has been carried out to foster compliance in Europe through the regulation of chemicals in various industries including the agricultural sector. However, more methodological work needs to examine and test the same studies in a new context, such as Africa. Thus comparing the types of exposure from a broad spectrum such as cultural diversity, food staples, food safety systems, and climatic variations in agricultural land.

Illnesses caused by chemical exposure in food need to be studied across all agriculture-intensive counties. Methods to robustly capture the economic impact of illnesses caused by chemical contamination in food need to be designed and aligned with global goals on sustainability and best practices. It would also serve a great purpose to involve the food industry in expressing their challenges regarding systems of producing food with minimal chemical contamination.

## Figures and Tables

**Table 2:** Summary of the classification of pesticides and human health effects.

Pesticide Name	Classification	Route of Exposure/Pathway	Documented Health Effects	References
Dichlorodiphenyltrichloroethane (DDT)	Organochlorine	Ingestion, inhalation-Crop fields	Parkinson’s disease, neurotoxic effects	[12,48,67,82]
Hexachlorocyclohexane	Organochlorine	Ingestion of contaminated food	Birth defects in humans	[89,90]
Benzene hexachloride	Organophosphates	Ingestion Locust control	Liver disease, skin lesion, loss of hair, thyroid damage, ulceration	[82,87]
Malathion, chlorpyrifos, diazinon, temephos	Organophosphates	Ingestion	Neurologic toxic effects, impaired vision, headache, dizziness	[14,41,55,57]

**Table 3:** Maximum levels for selected pesticides that may be present in foodstuffs as per the Codex Alimentarius International Food Standards [95].

Pesticide	Foodstuff	Maximum Residue Levels (MRL) (mg/kg)
Abamectin	Citrus fruits	0.02
	Soya beans (dry)	0.002
Acephate	Cabbages, head	2
	Meat (From mammals other than marine mammals	0.05
DDT	Carrot	0.2
	Cereal grains	0.1
	Poultry meat	0.3
Azoxystrobin	Strawberry	10
	Sunflower seed	0.5
	Banana	2
	Soya bean (dry)	0.5
	Sorghum	10
	Sugar cane	0.05
	Poultry meat	0.01
	Rice	5
Tebuconazole	Apples	1
	Apricot	2
	Barley	2
	Broccoli	0.2
	Carrot	0.4
	Coffee beans	0.1
	Prunes, dried	3
	Tomato	0.7
	Wheat	0.15

**Table 5:** Maximum levels of selected metals in foodstuffs as determined by in the Codex Alimentarius International Food Standards [127].

Metal	Foodstuff	ML (mg/kg)
Arsenic, Total (As-tot)	Edible fats and oils	0.1
	Olive oil, refined	0.1
	Margarine	0.1
	Vegetable oil, crude	0.1
Cadmium (Cd)	Brassica vegetables	0.05
	Bulb vegetables	0.05
	Fruiting vegetables (Excluding tomatoes & edible fungi)	0.05
	Leafy vegetables	0.2
	Legume vegetables	0.1
Tin (Sn)	Canned beverages	150
	Cooked cured chopped meat (Applies to products in containers other than tinplate containers)	50
	Cooked cured ham (Applies to products in containers other than tinplate containers)	50
	Corned beef (Applies to products in containers other than tinplate containers)	50
Lead (Pb)	Fruits, except berries and other small fruits (After removal of the stem, cap, stone, crown, and/or seeds but calculated on whole fruit	0.1
	Brassica vegetables	0.1
	Bulb vegetables	0.1
	Fruiting vegetables (Excluding fungi and mushrooms)	0.05
	Leafy vegetables	0.3
	Legume vegetables	0.1
	Canned fruits	0.1
	Canned vegetables (Excluding canned brassica vegetables)	0.1
	Fruit juices, nectars, and ready-to-drink fruit drinks/juices (Excluding juices and nectars from berries and small fruits and passion fruit juices	0.03
	Poultry, Edible offal of	0.5
	Fish (whole commodity or portions, without the viscera).	0.3
	Milk	0.02
	Secondary milk products (Products made from milk)	0.02
	Infant formula, a formula for special medical purposes intended for infants and follow-up formula	0.01

## References

[1] Ahmad S., Masood F., Khatoon K., Malik A., Malik A., Erginkaya Z., Erten H. (2019). Risk Management of Chemical Hazards Arising During Food Manufacturing. Health and Safety Aspects of Food Processing Technologies.

[2] Aiyar A., Pingali P. (2020). Pandemics and food systems—towards a proactive food safety approach to disease prevention & management. Food Secur..

[3] Vipham J.L., Amenu K., Alonso S., Ndahetuye J.-B., Zereyesus Y., Nishimwe K., Bowers E., Maier D., Sah K., Havelaar A. (2020). No food security without food safety: Lessons from livestock related research. Glob. Food Secur..

[4] Yu Z., Jung D., Park S., Hu Y., Huang H., Rasco B.A., Wang S., Ronholm J., Lu X., Chen J. (2020). Smart traceability for food safety. Crit. Rev. Food Sci. Nutr..

[5] Nerín C., Aznar M., Carrizo D. (2016). Food contamination during food process. Trends Food Sci. Technol..

[6] Niu B., Zhang H., Zhou G., Zhang S., Yang Y., Deng X., Chen Q. (2021). Safety risk assessment and early warning of chemical contamination in vegetable oil. Food Control.

[7] Bhalla T.C., Monika S.S., Singh R.L., Mondal S. (2019). International laws and food-borne illness. Food Safety and Human Health.

[8] Mandlate J.S., Soares B.M., Andrade C.F.F., Colling L.A., Primel E.G., Mesko M.F., Duarte F.A. (2020). Determination of trace elements in Sergio mirim: An evaluation of sample preparation methods and detection techniques. Environ. Sci. Pollut. Res..

[9] Shariatifar N., Seilani F., Jannat B., Nazmara S., Arabameri M. (2020). The concentration and health risk assessment of trace elements in commercial soft drinks from Iran marketed. Int. J. Environ. Anal. Chem..

[10] Hossain M., Karmakar D., Begum S.N., Ali S.Y., Patra P.K. (2021). Recent trends in the analysis of trace elements in the field of environmental research: A review. Microchem. J..

[11] My N.H.D., Demont M., Verbeke W. (2021). Inclusiveness of consumer access to food safety: Evidence from certified rice in Vietnam. Glob. Food Sec..

[12] Thakali A., MacRae J.D. (2021). A review of chemical and microbial contamination in food: What are the threats to a circular food system?. Environ. Res..

[13] Oliver M., Geniets A., Winters N., Rega I., Mbae S.M. (2015). What do community health workers have to say about their work, and how can this inform improved programme design? A case study with CHWs within Kenya. Glob. Health Action.

[14] Rather I.A., Koh W.Y., Paek W.K., Lim J. (2017). The sources of chemical contaminants in food and their health implications. Front. Pharmacol..

[15] Tempelhoff J.W.N. (2009). Civil society and sanitation hydropolitics: A case study of South Africa’s Vaal River Barrage. Phys. Chem. Earth.

[16] Ng C.A., von Goetz N. (2017). The global food system as a transport pathway for hazardous chemicals: The missing link between emissions and exposure. Environ. Health Perspect..

[17] Wang X., Wang C., Zhu T., Gong P., Fu J., Cong Z. (2019). Persistent organic pollutants in the polar regions and the Tibetan Plateau: A review of current knowledge and future prospects. Environ. Pollut..

[18] Ortiz-Solà J., Abadias M., Colás-Medà P., Sánchez G., Bobo G., Viñas I. (2020). Evaluation of a sanitizing washing step with different chemical disinfectants for the strawberry processing industry. Int. J. Food Microbiol..

[19] Bernardi A.O., Garcia M.V., Copetti M.V. (2019). Food industry spoilage fungi control through facility sanitization. Curr. Opin. Food Sci..

[20] Marriott N.G., Schilling M.W., Gravani R.B., Marriott N.G., Schilling M.W., Gravani R.B. (2018). Food Contamination Sources. Principles of Food Sanitation.

[21] Sharif M.K., Javed K., Nasir A., Holban M.A., Grumezescu A.M. (2018). Foodborne Illness: Threats and Control. Foodborne Diseases.

[22] Khare S., Tonk A., Rawat A. (2018). Foodborne diseases outbreak in India: A review. Int. J. Food Sci. Nutr..

[23] Mohammadzadeh-Aghdash H., Sohrabi Y., Mohammadi A., Shanehbandi D., Dehghan P., Dolatabadi J.E.N. (2018). Safety assessment of sodium acetate, sodium diacetate and potassium sorbate food additives. Food Chem..

[24] Jayant D., Halami P.M., Kataki R., Khanal S.K. (2020). Industrial perspective of food preservatives from microbial origin. Current Developments in Biotechnology and Bioengineering.

[25] Faustino M., Veiga M., Sousa P., Costa E.M., Silva S., Pintado M. (2019). Agro-food byproducts as a new source of natural food additives. Molecules.

[26] Hamid A.A., Risikat A., Abdulmumeen H.A., Risikat A.N., Sururah A.R. (2012). Composition and bioactivities of Essential Oils View project Food: Its preservatives, additives and applications. IJCBS.

[27] Roca-Saavedra P., Mendez-Vilabrille V., Miranda J.M., Nebot C., Cardelle-Cobas A., Franco C.M., Cepeda A. (2018). Food additives, contaminants and other minor components: Effects on human gut microbiota—A review. J. Physiol. Biochem..

[28] Kamal A.A., Fawzia S.E.S. (2018). Toxicological and safety assessment of tartrazine as a synthetic food additive on health biomarkers: A review. Afr. J. Biotechnol..

[29] Szűcs V., Szabó E., Guerrero L., Tarcea M., Bánáti D. (2019). Modelling of avoidance of food additives: A cross country study. Int. J. Food Sci. Nutr..

[30] Bruna G.O.L., Thais A.C.C., Lígia A.C.C. (2018). Food additives and their health effects: A review on preservative sodium benzoate. Afr. J. Biotechnol..

[31] Pasca C., Marghitas L.A., Bobis O., Dezmirean D.S., Margaoan R., Muresan C. (2014). Total content of polyphenols and antioxidant activity of different melliferous plants. Bull. UASVM Anim. Sci. Biotechnol..

[32] Martins F.C.O.L., Sentanin M.A., De Souza D. (2019). Analytical methods in food additives determination: Compounds with functional applications. Food Chem..

[33] Chiesa L.M., Zanardi E., Nobile M., Panseri S., Ferretti E., Ghidini S., Foschini S., Ianieri A., Arioli F. (2019). Food risk characterization from exposure to persistent organic pollutants and metals contaminating eels from an Italian lake. Food Addit. Contam. Part A Chem. Anal. Control. Expo. Risk Assess..

[34] Jansen T., Claassen L., van Kamp I., Timmermans D.R.M. (2020). ‘All chemical substances are harmful.’ public appraisal of uncertain risks of food additives and contaminants. Food Chem. Toxicol..

[35] Sha J.-B., Zhang S.-S., Lu Y.-M., Gong W.-J., Jiang X.-P., Wang J.-J., Qiao T.-L., Zhang H.-H., Zhao M.-Q., Wang D.-P. (2018). Effects of the long-term consumption of hydrogen-rich water on the antioxidant activity and the gut flora in female juvenile soccer players from Suzhou, China. Med. Gas Res..

[36] Zhong Y., Wu L., Chen X., Huang Z., Hu W. (2018). Effects of food-additive-information on consumers’ willingness to accept food with additives. Int. J. Environ. Res. Public Health.

[37] Bashir K.M.I., Kim J.S., An J.H., Sohn J.H., Choi J.S. (2017). Natural Food Additives and Preservatives for Fish-Paste Products: A Review of the Past, Present, and Future States of Research. J. Food Qual..

[38] Franco R., Navarro G., Martínez-Pinilla E. (2019). Antioxidants versus food antioxidant additives and food preservatives. Antioxidants.

[39] Muncke J., Andersson A.-M., Backhaus T., Boucher J.M., Almroth B.C., Castillo A., Chevrier J., Demeneix B.A., Emmanuel J.A., Fini J.-B. (2020). Impacts of food contact chemicals on human health: A consensus statement. Environ. Health Glob. Access Sci. Source.

[40] Mania I., Delgado A.M., Barone C., Parisi S. (2018). Food Packaging and the Mandatory Traceability in Europe. Traceability in the Dairy Industry in Europe: Theory and Practice.

[41] Han J.W., Ruiz-Garcia L., Qian J.P., Yang X.T. (2018). Food Packaging: A Comprehensive Review and Future Trends. Compr. Rev. Food Sci. Food Saf..

[42] Deshwal G.K., Panjagari N.R. (2020). Review on metal packaging: Materials, forms, food applications, safety and recyclability. J. Food Sci. Technol..

[43] Sofi S.A., Singh J., Rafiq S., Ashraf U., Dar B.N., Nayik G.N. (2018). A Comprehensive Review on Antimicrobial Packaging and its Use in Food Packaging. Curr. Nutr. Food Sci.

[44] Geueke B., Groh K., Muncke J. (2018). Food packaging in the circular economy: Overview of chemical safety aspects for commonly used materials. J. Clean. Prod..

[45] Guerreiro T.M., de Oliveira D.N., Melo C.F.O.R., de Oliveira Lima E., Catharino R.R. (2018). Migration from plastic packaging into meat. Food Res. Int..

[46] Ernstoff A., Niero M., Muncke J., Trie X., Rosenbaum R.K., Hauschild M., Fantke P. (2019). Challenges of including human exposure to chemicals in food packaging as a new exposure pathway in life cycle impact assessment. Int. J. Life Cycle Assess.

[47] Karmaus A.L., Osborn R., Krishan M. (2018). Scientific advances and challenges in safety evaluation of food packaging materials: Workshop proceedings. Regul. Toxicol. Pharmacol..

[48] Bari M.L., Yeasmin S., Grumezescu A.M., Alina Maria H. (2018). Foodborne Diseases and Responsible Agents. Food Safety and Preservation.

[49] Thompson L.A., Darwish W.S. (2019). Environmental Chemical Contaminants in Food: Review of a Global Problem. J. Toxicol..

[50] Dainelli D., Gontard N., Spyropoulos D., Zondervan-van den Beuken E., Tobback P. (2008). Active and intelligent food packaging: Legal aspects and safety concerns. Trends Food Sci. Technol..

[51] Ahmed I., Lin H., Zou L., Li Z., Brody A.L., Qazi I.M., Lv L., Pavase T.R., Khan M.U., Khan S. (2018). An overview of smart packaging technologies for monitoring safety and quality of meat and meat products. Packag. Technol. Sci..

[52] Hahladakis J.N., Velis C.A., Weber R., Iacovidou E., Purnell P. (2018). An overview of chemical additives present in plastics: Migration, release, fate and environmental impact during their use, disposal and recycling. J. Hazard. Mater..

[53] Mastromatteo M., Mastromatteo M., Conte A., Del Nobile M.A. (2010). Advances in controlled release devices for food packaging applications. Trends Food Sci. Technol..

[54] Fred-Ahmadu O.H., Bhagwat G., Oluyoye I., Benson N.U., Ayejuyo O.O., Palanisami T. (2020). Interaction of chemical contaminants with microplastics: Principles and perspectives. Sci. Total Environ..

[55] Deng Z.H., Li N., Jiang H.L., Lin J.M., Zhao R.S. (2019). Pretreatment techniques and analytical methods for phenolic endocrine disrupting chemicals in food and environmental samples. TrAC Trends Anal. Chem..

[56] Bajpai V.K., Kamle M., Shukla S., Mahato D.K., Chandra P., Hwang S.K., Kumar P., Huh Y.S., Han Y.-K. (2018). Prospects of using nanotechnology for food preservation, safety, and security. J. Food Drug Anal..

[57] Ramos Ó.L., Pereira R.N., Cerqueirat M.M., Martins J.R., Teixeira J.G., Malcata F.X., Vicente A.A., Grumezescu A.M., Holban A.M. (2018). Bio-based Nanocomposites for food Packaging and Their Effect in Food Quality and Safety. Food Packaging and Preservation.

[58] Deshwal G.K., Panjagari N.R., Alam T. (2019). An overview of paper and paper based food packaging materials: Health safety and environmental concerns. J. Food Sci. Technol..

[59] Pilevar Z., Bahrami A., Beikzadeh S., Hosseini H., Jafari S.M. (2019). Migration of styrene monomer from polystyrene packaging materials into foods: Characterization and safety evaluation. Trends Food Sci. Technol..

[60] Adeyeye S.A.O. (2019). Food packaging and nanotechnology: Safeguarding consumer health and safety. Nutr. Food Sci..

[61] Janjarasskul T., Suppakul P. (2018). Active and intelligent packaging: The indication of quality and safety. Crit. Rev. Food Sci. Nutr..

[62] Yucel U. (2016). Intelligent Packaging.

[63] Majid I., Ahmad Nayik G., Mohammad Dar S., Nanda V. (2018). Novel food packaging technologies: Innovations and future prospective. J. Saudi Soc. Agric. Sci..

[64] Galstyan V., Bhandari M.P., Sberveglieri V., Sberveglieri G., Comini E. (2018). Metal oxide nanostructures in food applications: Quality control and packaging. Chemosensors.

[65] Freeman S. (2018). Plastic food contact articles—Food chemical safety unwrapped. Environ. Health Rev..

[66] de Fátima Poças M., Hogg T. (2007). Exposure assessment of chemicals from packaging materials in foods: A review. Trends Food Sci. Technol..

[67] Deb P., Holban A.M., Grumezescu A.M. (2018). Environmental Pollution and the burden of Food-Borne Diseases. Foodborne Diseases.

[68] Pius C., Sichilongo K., Koosaletse Mswela P., Dikinya O. (2019). Monitoring polychlorinated dibenzo-p-dioxins/dibenzofurans and dioxin-like polychlorinated biphenyls in Africa since the implementation of the Stockholm Convention—An overview. Environ. Sci. Pollut. Res..

[69] Bruce-Vanderpuije P., Megson D., Reiner E.J., Bradley L., Adu-Kumi S., Gardella J.A. (2019). The state of POPs in Ghana—A review on persistent organic pollutants: Environmental and human exposure. Environ. Pollut..

[70] Gaur N., Narasimhulu K., PydiSetty Y. (2018). Recent advances in the bio-remediation of persistent organic pollutants and its effect on environment. J. Clean. Prod..

[71] Volschenk C.M., Gerber R., Mkhonto R.T., Ikenaka Y., Yohannes Y.B., Nakayama S., Ishizuka M., van Vuren J.H.J., Wepener V., Smit N.J. (2019). Bioaccumulation of persistent organic pollutants and their trophic transfer through the food web: Human health risks to the rural communities reliant on fish from South Africa’s largest floodplain. Sci. Total Environ..

[72] Guo W., Pan B., Sakkiah S., Yavas G., Ge W., Zou W., Tong W., Hong H. (2019). Persistent organic pollutants in food: Contamination sources, health effects and detection methods. Int. J. Environ. Res. Public Health.

[73] Loganathan B.G., Masunaga S., Gupta R.C. (2020). PCBs, dioxins, and furans: Human exposure and health effects. Handbook of Toxicology of Chemical Warfare Agents.

[74] Kang Y., Cao S., Yan F., Qin N., Wang B., Zhang Y., Shao K., El-Maleh C.A., Duan X. (2020). Health risks and source identification of dietary exposure to indicator polychlorinated biphenyls (PCBs) in Lanzhou, China. Environ. Geochem. Health.

[75] Rusin M., Dziubanek G., Marchwińska-Wyrwał E., Ćwieląg-Drabek M., Razzaghi M., Piekut A. (2019). PCDDs, PCDFs and PCBs in locally produced foods as health risk factors in Silesia Province, Poland. Ecotoxicol. Environ. Saf..

[76] Ssebugere P., Sillanpää M., Matovu H., Mubiru E. (2019). Human and environmental exposure to PCDD/Fs and dioxin-like PCBs in Africa: A review. Chemosphere.

[77] Kumar M., Chand R., Shah K., Patra J.K., Gitishree D., Shin H.-S. (2018). Mycotoxins and pesticides: Toxicity and applications in food and feed. Microbial Biotechnology.

[78] Zikankuba V.L., Mwanyika G., Ntwenya J.E., James A. (2019). Pesticide regulations and their malpractice implications on food and environment safety. Cogent Food Agric..

[79] Kosamu I., Kaonga C., Utembe W. (2020). A critical review of the status of pesticide exposure management in Malawi. Int. J. Environ. Res. Public Health.

[80] Tudi M., Ruan H.D., Wang L., Lyu J., Sadler R., Connell D., Chu C., Phung D.T. (2021). Agriculture development, pesticide application and its impact on the environment. Int. J. Environ. Res. Public Health.

[81] Xu Y., Wang H., Li X., Zeng X., Du Z., Cao J., Jiang W. (2021). Metal–organic framework for the extraction and detection of pesticides from food commodities. Compr. Rev. Food Sci. Food Saf..

[82] Taiwo A.M. (2019). A review of environmental and health effects of organochlorine pesticide residues in Africa. Chemosphere.

[83] Sharma A., Kumar V., Shahzad B., Tanveer M., Sidhu G.P.S., Handa N., Kohli S.K., Yadav P., Bali A.S., Parihar R.D. (2019). Worldwide pesticide usage and its impacts on ecosystem. SN Appl. Sci..

[84] Liao H., Tang M., Luo L., Li C., Chiclana F., Zeng X.-J. (2018). A bibliometric analysis and visualization of medical big data research. Sustainability.

[85] Chiocchetti G.M., Jadán-Piedra C., Monedero V., Zúñiga M., Vélez D., Devesa V. (2019). Use of lactic acid bacteria and yeasts to reduce exposure to chemical food contaminants and toxicity. Crit. Rev. Food Sci. Nutr..

[86] Massoud R., Hadiani M.R., Hamzehlou P., Khosravi-Darani K. (2019). Bioremediation of heavy metals in food industry: Application of Saccharomyces cerevisiae. Electron. J. Biotechnol..

[87] Santarelli G.A., Migliorati G., Pomilio F., Marfoglia C., Centorame P., D’Agostino A., D’Aurelio R., Scarpone R., Battistelli N., Di Simone F. (2018). Assessment of pesticide residues and microbial contamination in raw leafy green vegetables marketed in Italy. Food Control.

[88] Raymundo-Pereira P.A., Gomes N.O., Shimizu F.M., Machado S.A.S., Oliveira O.N. (2021). Selective and sensitive multiplexed detection of pesticides in food samples using wearable, flexible glove-embedded non-enzymatic sensors. Chem. Eng. J..

[89] Islam M.A., Ullah A., Habib M., Chowdhury M.T.I., Khan M.S.I., Kaium A., Prodhan M.D.H. (2019). Determination of Major Organophosphate Insecticide Residues in Cabbage Samples From Different Markets of Dhaka. Asia Pac. Environ. Occup. Health J..

[90] Margenat A., Matamoros V., Díez S., Cañameras N., Comas J., Bayonaa M.B. (2019). Occurrence and human health implications of chemical contaminants in vegetables grown in peri-urban agriculture. Environ. Int..

[91] Mazzoni M., Boggio E., Manca M., Piscia R., Quadroni S., Bellasi A., Bettinetti R. (2018). Trophic transfer of persistent organic pollutants through a pelagic food web: The case of Lake Como (Northern Italy). Sci. Total Environ..

[92] European Food Safety Authority (2011). Scientific support for preparing an EU position in the 43rd Session of the Codex Committee on Pesticide Residues (CCPR). EFSA J..

[93] European Food Safety Authority (2019). Scientific support for preparing an EU position in the 51st Session of the Codex Committee on Pesticide Residues (CCPR). EFSA J..

[94] Nasreddine L., Parent-Massin D. (2002). Food contamination by metals and pesticides in the European Union. Should we worry?. Toxicol. Lett..

[95] Codex Alimentarius International Food Standards. http://www.fao.org/fao-who-codexalimentarius/codex-texts/dbs/pestres/pesticides/en/.

[96] Kirchhelle C. (2018). Pharming animals: A global history of antibiotics in food production (1935–2017). Palgrave Commun..

[97] Baynes R.E., Dedonder K., Kissell L., Mzyk D., Marmulak T., Smith G., Tell L., Gehring R., Davis J., Riviere J.E. (2016). Health concerns and management of select veterinary drug residues. Food Chem. Toxicol..

[98] Zhao L., Lucas D., Long D., Richter B., Stevens J. (2018). Multi-class multi-residue analysis of veterinary drugs in meat using enhanced matrix removal lipid cleanup and liquid chromatography-tandem mass spectrometry. J. Chromatogr. A.

[99] Bacanlı M., Başaran N. (2019). Importance of antibiotic residues in animal food. Food Chem. Toxicol..

[100] Wang H., Rena L., Yua X., Hua J., Chen Y., He G., Jianga Q. (2017). Antibiotic residues in meat, milk and aquatic products in Shanghai and human exposure assessment. Food Control.

[101] Njoga E.O., Onunkwo J.I., Okoli C.E., Ugwuoke W.I., Nwanta J.A., Chah K.F. (2018). Assessment of antimicrobial drug administration and antimicrobial residues in food animals in Enugu State, Nigeria. Trop. Anim. Health Prod..

[102] Hu Y., Cheng H. (2016). Health risk from veterinary antimicrobial use in China’s food animal production and its reduction. Environ. Pollut..

[103] Scott A.M., Beller B., Glasziou P., Clark J., Ranakusuma R.W., Byambasuren O., Bakhit M., Page S.W., Trott D., Mar C.D. (2018). Is antimicrobial administration to food animals a direct threat to human health? A rapid systematic review. Int. J. Antimicrob. Agents.

[104] Sivagami K., Vignesh V.J., Srinivasan R., Divyapriya G., Nambi I.M. (2020). Antibiotic usage, residues and resistance genes from food animals to human and environment: An Indian scenario. J. Environ. Chem. Eng..

[105] Moudgil P., Bedi J.S., Moudgil A.D., Gill J.P.S., Aulakh R.S. (2018). Emerging issue of antibiotic resistance from food producing animals in India: Perspective and legal framework. Food Rev. Int..

[106] Van T.T.H., Yidana Z., Smooker P.M., Coloe P.J. (2020). Antibiotic use in food animals worldwide, with a focus on Africa: Pluses and minuses. J. Glob. Antimicrob. Resist..

[107] Ronquillo G.M., Hernandez A.J.C. (2017). Antibiotic and synthetic growth promoters in animal diets: Review of impact and analytical methods. Food Control.

[108] Manage P.M. (2018). Heavy use of antibiotics in aquaculture: Emerging human and animal health problems—A review. SRI Lanka J. Aquat. Sci..

[109] Kang H.-S., Lee S.-B., Shin D., Jeong J., Hong J.-H., Rhee G.-S. (2018). Occurrence of veterinary drug residues in farmed fishery products in South Korea. Food Control.

[110] Mehdi Y., Létourneau-Montminy M.-P., Gaucher M.-L., Chorfi Y., Suresh G., Rouissi T., Brar S.K., Côté C., Ramirez A.A., Godbout S. (2018). Use of antibiotics in broiler production: Global impacts and alternatives. Anim. Nutr..

[111] Novak R. (2011). Are pleuromutilin antibiotics finally fit for human use?. Ann. N. Y. Acad. Sci..

[112] Bagheri A.R., Ghaedi M. (2020). Magnetic metal organic framework for pre-concentration of ampicillin from cow milk samples. J. Pharm. Anal..

[113] Tenenbein M.S., Tenenbein M. (2005). Acute pancreatitis due to erythromycin overdose. Pediatr. Emerg. Care.

[114] Kuppusamy S., Kakarla D., Venkateswarlu K., Megharaj M., Yoon Y.-E., Lee Y.B. (2018). Veterinary antibiotics (VAs) contamination as a global agro-ecological issue: A critical view. Agric. Ecosyst. Environ..

[115] Antoniadis V., Golia E.E., Liu Y.-T., Wang S.-L., Shaheen S.M., Rinklebe J. (2019). Soil and maize contamination by trace elements and associated health risk assessment in the industrial area of Volos, Greece. Environ. Int..

[116] Setia R., Dhaliwal S.S., Singh R., Kumar V., Taneja S., Kukal S.S., Pateriya B. (2021). Phytoavailability and human risk assessment of heavy metals in soils and food crops around Sutlej river, India. Chemosphere.

[117] Al-Othman Z.A., Ali R., Al-Othman A.M., Ali J., Habila A.M. (2016). Assessment of toxic metals in wheat crops grown on selected soils, irrigated by different water sources. Arab. J. Chem..

[118] Rai P.K., Lee S.S., Zhang M., Tsang Y.F., Kim K.H. (2019). Heavy metals in food crops: Health risks, fate, mechanisms, and management. Environ. Int..

[119] De Tandt E., Demuytere C., Van Asbroeck E., Moerman H., Mys N., Vyncke G., Delva L., Vermeulen A., Ragaert P., De meester S. (2021). A recycler’s perspective on the implications of REACH and food contact material (FCM) regulations for the mechanical recycling of FCM plastics. Waste Manag..

[120] Marini M., Angouria-Tsorochidou E., Caro D., Thomsen M. (2021). Daily intake of heavy metals and minerals in food—A case study of four Danish dietary profiles. J. Clean. Prod..

[121] Hejna M., Onelli E., Moscatelli A., Bellotto M., Cristiani C., Stroppa N., Rossi L. (2021). Heavy-metal phytoremediation from livestock wastewater and exploitation of exhausted biomass. Int. J. Environ. Res. Public Health.

[122] Mihaileanu R.G., Neamtiu I.A., Fleming M., Pop C., Bloom M.S., Roba C., Surcel M., Stamatian F., Gurzau E. (2019). Assessment of heavy metals (total chromium, lead, and manganese) contamination of residential soil and homegrown vegetables near a former chemical manufacturing facility in Tarnaveni, Romania. Environ. Monit. Assess..

[123] Afonne O.J., Ifediba E.C. (2020). Heavy metals risks in plant foods—Need to step up precautionary measures. Curr. Opin. Toxicol..

[124] Khezerlou A., Dehghan P., Moosavy M.H.., Kochakkhani H. (2020). Assessment of Heavy Metal Contamination and the Probabilistic Risk via Salad Vegetable Consumption in Tabriz, Iran. Biol. Trace Elem. Res..

[125] Edogbo B., Okolocha E., Maikai B., Aluwong T., Uchendu C. (2020). Risk analysis of heavy metal contamination in soil, vegetables and fish around Challawa area in Kano State, Nigeria. Sci. Afr..

[126] Misihairabgwi J.M., Ezekiel C.N., Sulyok M., Shephard G.S., Krska R. (2019). Mycotoxin contamination of foods in Southern Africa: A 10-year review (2007–2016). Crit. Rev. Food Sci. Nutr..

[127] Codex Alimentarius International Food Standards. http://www.fao.org/fileadmin/user_upload/livestockgov/documents/1_CXS_193e.pdf.

[128] Chen H., Liu S., Chen Y., Chen C., Yang H., Chen Y. (2020). Food safety management systems based on ISO 22000: 2018 methodology of hazard analysis compared to ISO 22000:2005. Accredit. Qual. Assur..

[129] Yapp C., Fairman R. (2006). Factors affecting food safety compliance within small and medium-sized enterprises: Implications for regulatory and enforcement strategies. Food Control.

[130] Crépet A., Luong T.H., Baines J., Boond D.E., Ennis J., Kennedy M., Massarelli I., Miller D., Nako S., Reuss R. (2021). An international probabilistic risk assessment of acute dietary exposure to pesticide residues in relation to codex maximum residue limits for pesticides in food. Food Control.

[131] Ambrus Á., Yang Y.Z. (2016). Global Harmonization of Maximum Residue Limits for Pesticides. J. Agric. Food Chem.

[132] Delatour T., Racault L., Bessaire T., Desmarchelier A. (2018). Screening of veterinary drug residues in food by LC-MS/MS. Background and challenges. Food Addit. Contam. Part A Chem. Anal. Control. Expo. Risk Assess..

[133] Boatemaa S., Barney M., Drimie S., Harper J., Korsten L., Pereira L. (2019). Awakening from the listeriosis crisis: Food safety challenges, practices and governance in the food retail sector in South Africa. Food Control.

